# Supplementary material for: Glycerol-based deep eutectic solvents for efficient and reversible iodine uptake from vapour phase
Source: Commun Chem. 2025 Jun 7;8:178. doi: 10.1038/s42004-025-01575-2 (PMC12145444; doi:10.1038/s42004-025-01575-2)
Supplement: Supplementary file 2 — Supplementary information [file 42004_2025_1575_MOESM2_ESM.pdf]

Supplementary information

Glycerol-based deep eutectic solvents for efficient and reversible iodine uptake from vapour phase

Daniele Motta,[a,z] Saïd Mondahchouo,[b,z] Stefano Nejrotti,[a,c] Carlotta Pontremoli,[a] Claudia Barolo,[a,c] Alessandro Damin,[a,*] and Matteo Bonomo[a,d,*]

[a] Department of Chemistry, NIS Interdepartmental Centre and INSTM Reference Centre, University of Turin, Via G. Quarello 15A, 10135 Turin, Italy

[b] Laboratory of Analytical Electrochemistry and Materials Engineering, University of Yaoundé I, P.O. Box 812, Yaoundé, Cameroon

[c] Institute of Science, Technology and Sustainability for Ceramics (ISSMC-CNR), Via Granarolo 64, 48018 Faenza, Italy

[d] Department of Basic and Applied Science for Engineering, La Sapienza University of Rome, Via A. Scarpa 10, 00178 Rome, Italy

alessandro.damin@unito.it and matteo.bonomo@uniroma1.it

[z] These authors contributed equally

[*] These authors jointly supervised this work

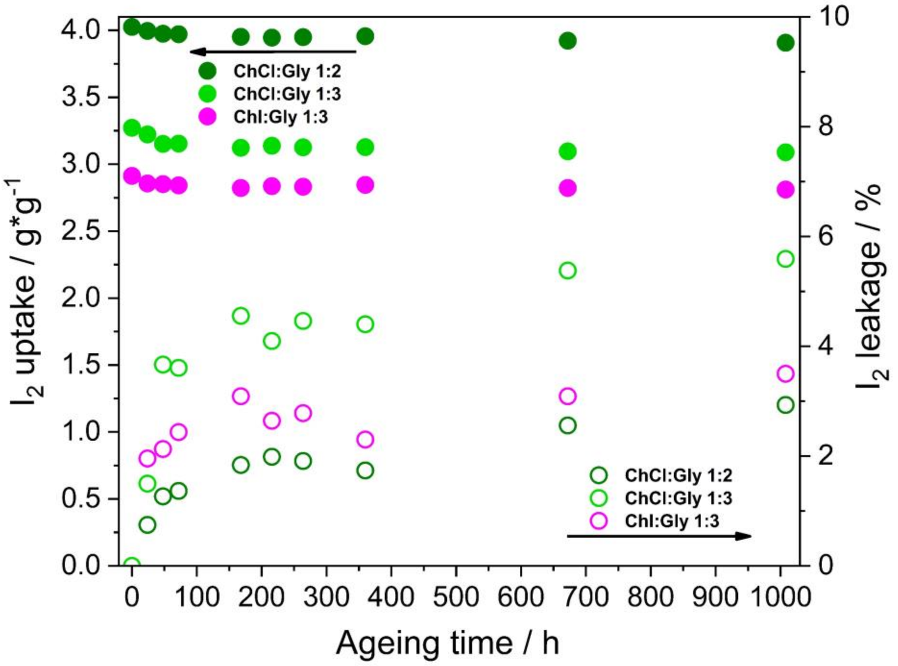

Supplementary Figure 1: stability over 42 days of the iodine-loaded DESs.

Supplementary Table 1. Some of the best performing adsorbents for vapour iodine uptake.

Adsorbent	Uptake efficiency / $g*g^{-1}$	Phase	Saturation time / h	T / °C	Ref. (main text)
**COF-TFPA**	8.61[A]	Vapour	96	75	**1**
**COF-TAPB**	7.94[A]	Vapour	96	75	**1**
**TJNU-203**	5.88[A]	Vapour	48	77	**2**
**TAPB-QOT COP**	4.64[A]	Vapour	24	77	**3**
**HCMP**	3.36[B]	Vapour	0.75	85	**4**
**Ionic-POP**	7.00[A]	Vapour	24	70	**5**
**[N_{1111}][AT]/MF**	6.94[A,B]	Vapour	30	75	**6**
**[Ch][Im]**	17.5[C]	Vapour	12	100	**7**
**[Ch][2-MIm]**	15.8[C]	Vapour	12	100	**7**
**DES1**	3.86[B]	Vapour	24	80	**This work**
**DES2**	3.02[B]	Vapour	24	80	**This work**
**DES3**	2.83[B]	Vapour	24	80	**This work**

[A] Regenerated in solution (methanol, ethanol, …)
[B] Reversible after heating
[C] Non reversible

Supplementary Table 2: fit parameters of the gravimetric iodine uptake curves.

Model	Asymptotic			
Equation	y = a-b*c^x			
Plot	DES1	DES2	DES3	GLY
a	3.8618 ± 0.1145	3.0204 ± 0.0987	2.8276 ± 0.1067	0.1749 ± 0.0077
b	3.5460 ± 0.1340	2.8342 ± 0.1177	2.5865 ± 0.1185	0.1452 ± 0.0160
c	0.0866 ± 0.0195	0.7932 ± 0.0230	0.8377 ± 0.0200	0.2965 ± 0.0951
R-Square	0.9808	0.9769	0.9738	0.8603

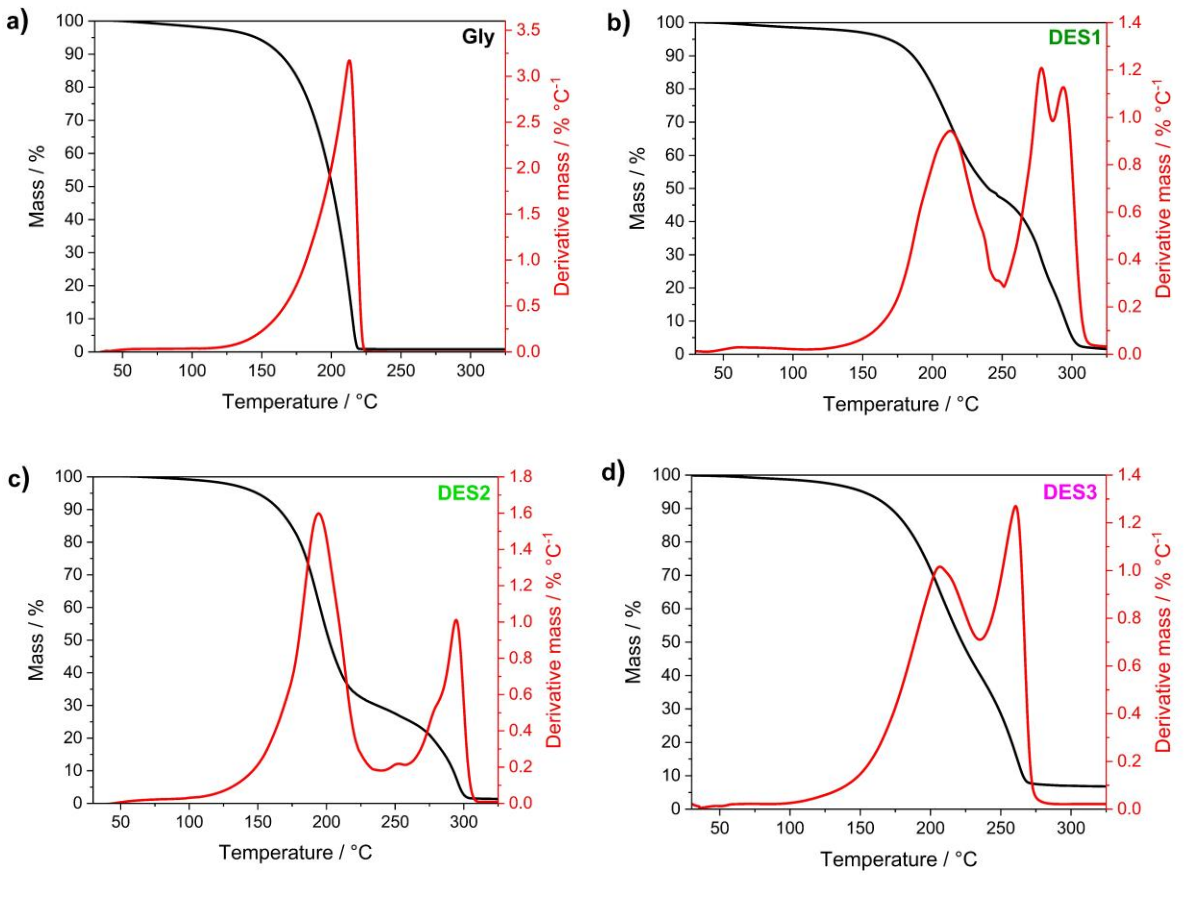

Supplementary Figure 2: TGA (black curves) and DTG (red curves) analyses of pure **a)** glycerol **b)** DES1 **c)** DES2 **d)** DES3.

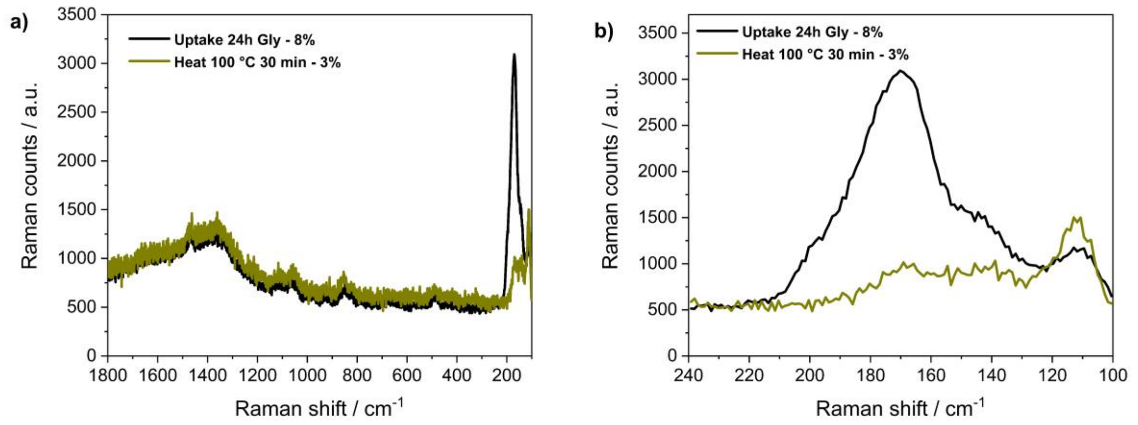

Supplementary Figure 3: Raman spectra of glycerol after the iodine uptake and after the heating at 100 °C for 30 minutes **a)** between 100 and 1800 cm^{-1} **b)** between 100 and 240 cm^{-1}.

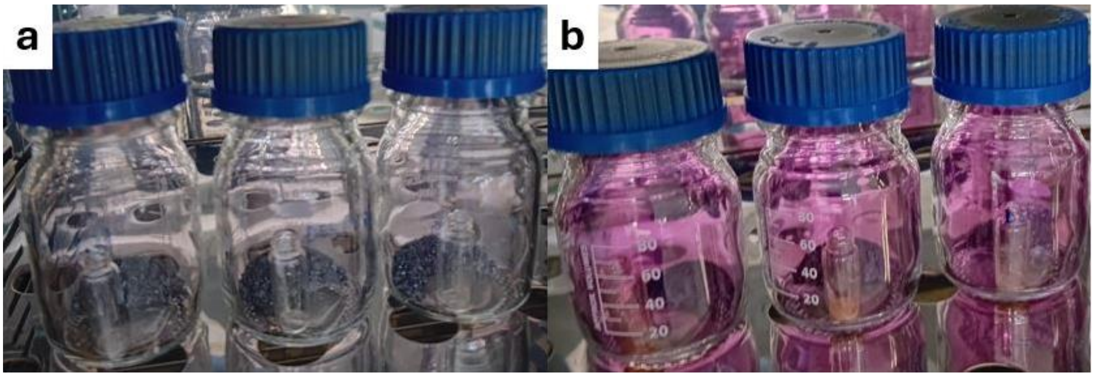

Supplementary Figure 4. Photographs of the experimental set-up **a)** before uptake and **b)** during the uptake in the oven at 80 °C.

Supplementary References

1. Xie, Y. *et al.* Efficient and simultaneous capture of iodine and methyl iodide achieved by a covalent organic framework. *Nat. Commun.* **13**, 1–10 (2022).
2. Zhang, L. *et al.* High iodine uptake in two-dimensional covalent organic frameworks. *Chem. Commun.* **57**, 5558–5561 (2021).
3. Yildirim, O. *et al.* Quinoid-Thiophene-Based Covalent Organic Polymers for High Iodine Uptake: When Rational Chemical Design Counterbalances the Low Surface Area and Pore Volume. *ACS Appl. Mater. Interfaces* **15**, 15819–15831 (2023).
4. Liao, Y., Weber, J., Mills, B. M., Ren, Z. & Faul, C. F. J. Highly Efficient and Reversible Iodine Capture in Hexaphenylbenzene-Based Conjugated Microporous Polymers. *Macromolecules* **49**, 6322–6333 (2016).
5. Sen, A. *et al.* Functionalized Ionic Porous Organic Polymers Exhibiting High Iodine Uptake from Both the Vapor and Aqueous Medium. *ACS Appl. Mater. Interfaces* **13**, 34188–34196 (2021).
6. Guo, S. J. *et al.* Efficient capture of iodine by charge-induced effect of nitrogen-rich ionic liquids. *Chem. Eng. J.* **475**, 146221 (2023).
7. Li, R. *et al.* Imidazolate ionic liquids for high-capacity capture and reliable storage of iodine. *Commun. Chem.* **1**, 1–8 (2018).
