# Supplementary material for: Glycerol-based deep eutectic solvents for efficient and reversible iodine uptake from vapour phase
Source: Commun Chem. 2025 Jun 7;8:178. doi: 10.1038/s42004-025-01575-2 (PMC12145444; doi:10.1038/s42004-025-01575-2)
Supplement: Supplementary file 3 — Description of Additional Supplementary Files [file 42004_2025_1575_MOESM3_ESM.pdf]

Description of Additional Supplementary Files

File name: Supplementary Data 1

Description: Summary of average iodine uptake values from gravimetric measurements.

File name: Supplementary Data 2

Description: Iodine uptake data from the three gravimetric trials.
